# Supplementary material for: Iron Oxide–Silica Core–Shell Nanoparticles Functionalized with Essential Oils for Antimicrobial Therapies
Source: Antibiotics (Basel). 2021 Sep 21;10(9):1138. doi: 10.3390/antibiotics10091138 (PMC8467872; doi:10.3390/antibiotics10091138)
Supplement: Supplementary file 1 [file antibiotics-10-01138-s001.zip › antibiotics-1355216-supplementary.pdf]

Supplementary Materials

Iron Oxide-Silica Core-Shell Nanoparticles Functionalized with Essential Oils for Antimicrobial Therapies

Figures S1 and S2 represent the size distribution and the correlation graphs, respectively, for the samples synthesized through the co-precipitation method, i.e., Fe_3O_4@SiO_2_CP, Fe_3O_4@SiO_2@thyme_CP, Fe_3O_4@SiO_2@rosemary_CP, and Fe_3O_4@SiO_2@basil_CP.

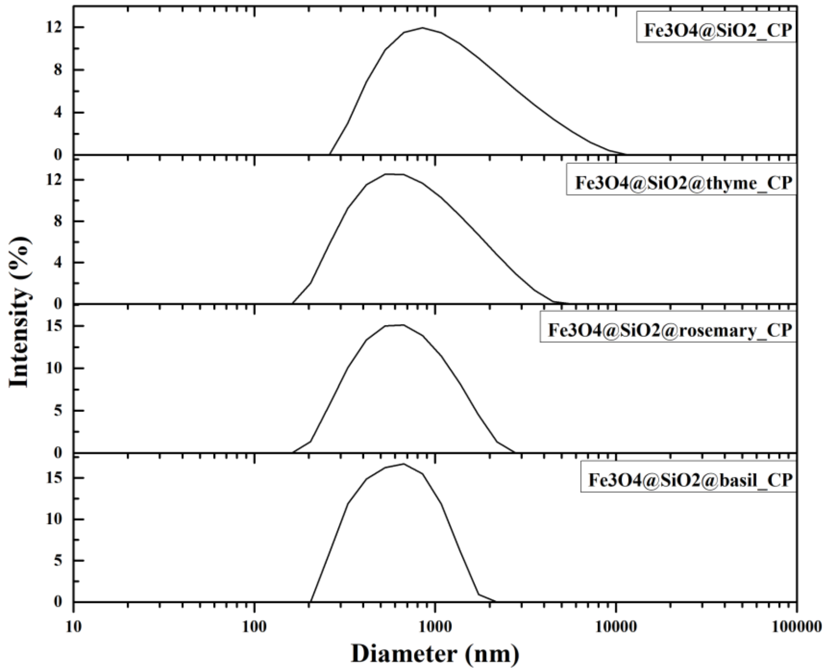

Figure S1: Size distribution for samples Fe_3O_4@SiO_2_CP, Fe_3O_4@SiO_2@thyme_CP, Fe_3O_4@SiO_2@rosemary_CP, and Fe_3O_4@SiO_2@basil_CP.

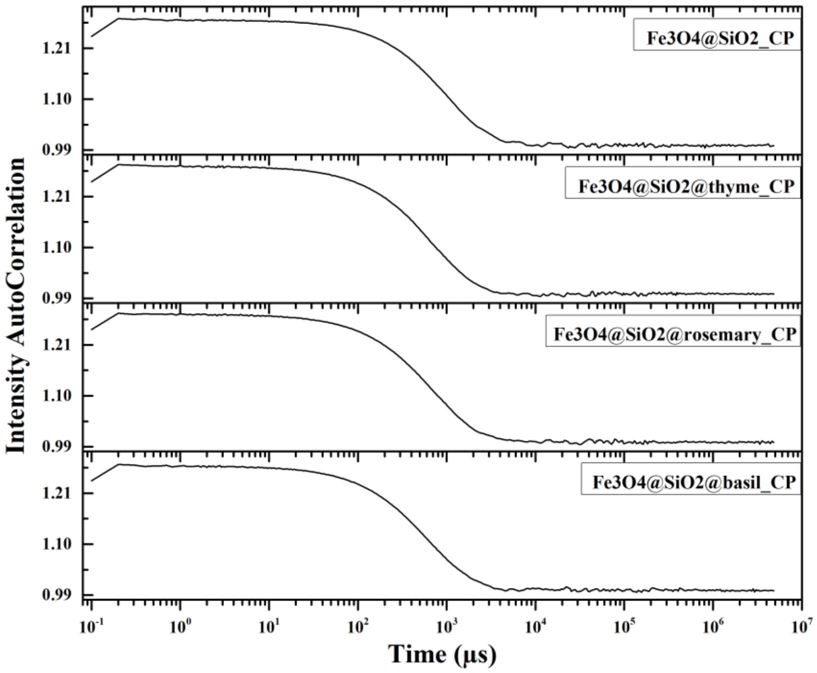

Figure S2: Correlation graphs for samples $Fe_3O_4@SiO_2$_CP, $Fe_3O_4@SiO_2$@thyme_CP, $Fe_3O_4@SiO_2$@rosemary_CP, and $Fe_3O_4@SiO_2$@basil_CP.

Figures 3 and 4 represent the size distribution and the correlation graphs, respectively, for the samples synthesized through the co-precipitation method, i.e., $Fe_3O_4@SiO_2$_SW, $Fe_3O_4@SiO_2$@thyme_SW, $Fe_3O_4@SiO_2$@rosemary_SW, and $Fe_3O_4@SiO_2$@basil_SW.

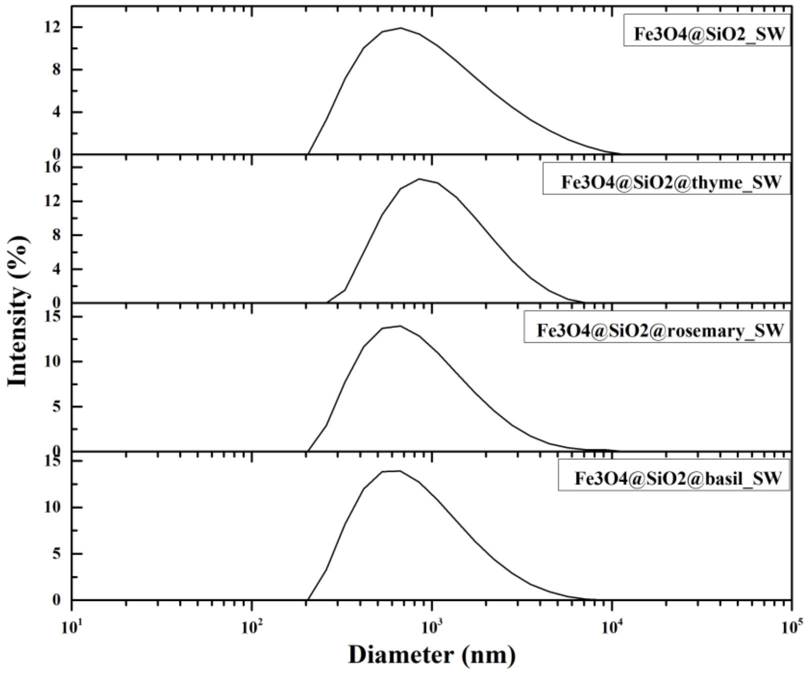

Figure S3: Size distribution for samples $Fe_3O_4@SiO_2$_CP, $Fe_3O_4@SiO_2$@thyme_CP, $Fe_3O_4@SiO_2$@rosemary_CP, and $Fe_3O_4@SiO_2$@basil_CP.

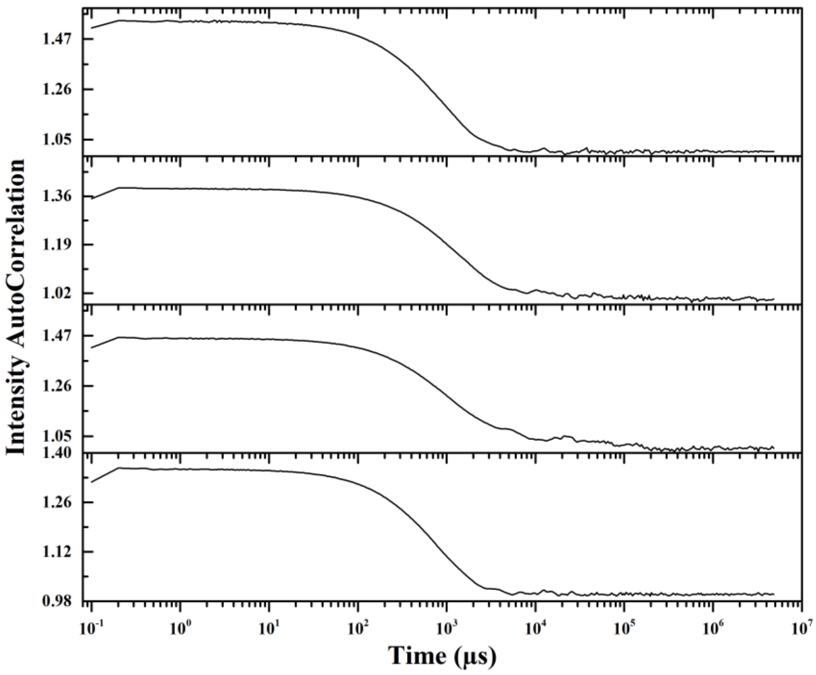

Figure S4: Correlation graphs for samples Fe_3O_4@SiO_2_CP, Fe_3O_4@SiO_2@thyme_CP, Fe_3O_4@SiO_2@rosemary_CP, and Fe_3O_4@SiO_2@basil_CP.

The compounds identified within EOs and their retention time were summarized in Table S1.

Table S1: The compounds identified within thyme, rosemary, and basil EOs, and their retention time.

Sample	Compound	Retention time
thyme EO	α-pinene	9.467
	camphene	10.050
	sabinene	11.051
	β-pinene	12.520
	α-phellandrene	12.114
	1,4-cineol	12.447
	p-mentha-1,5,8-triene	12.780
	d-limonene	12.978
	eucalyptol	13.083
	γ-terpinene	14.052
	terpinolene	15.062
	linalool	15.656
	fenchol	16.323
	1-terpineol	19.147
	endo-borneol	18.272
	4-terpineol	18.595
	α-copaene	25.305
	caryophyllene	26.681
	m-camphorene	41.436
rosemary EO	camphene	10.050
	fenchene	11.050
	β-myrcene	11.509
	o-cymene	12.801
	eucalyptol	13.061
	3-thujene	14.041
	linalool	15.604
	(+)-2-bornanone	17.323
	endo-borneol	18.261
	α-terpineol	19.095
	(-)-bornyl acetate	22.231
	α-copaene	25.305
	caryophyllene	26.722
	humulene	27.848
	caryophyllene oxide	31.641
basil EO	α-pinene	9.467

	camphene	10.050
	sabinene	11.051
	β-pinene	12.520
	α-phellandrene	12.114
	1,4-cineol	12.447
	p-mentha-1,5,8-triene	12.780
	d-limonene	12.978
	eucalyptol	13.083
	γ-terpinene	14.052
	terpinolene	15.062
	linalool	15.656
	fenchol	16.323
	1-terpineol	19.147
	endo-borneol	18.272
	4-terpineol	18.595
	α-copaene	25.305
	caryophyllene	26.681
	m-camphorene	41.436

The chromatogram profiles for each EO and EOs-functionalized core-shell nanoparticles and the mass spectra for each of the major compounds identified within the EOs can be found in the Supplementary Material.

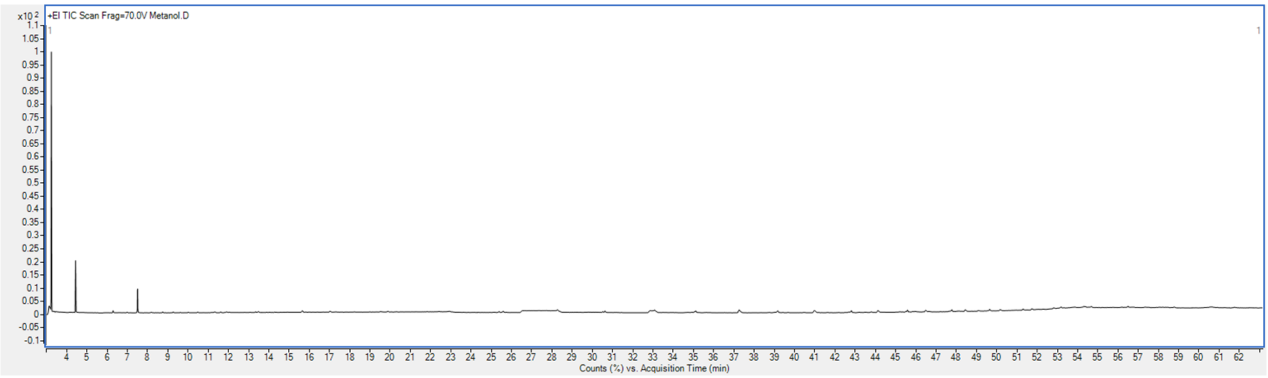

Figure S5: Chromatographic profile of the extraction solvent (methanol).

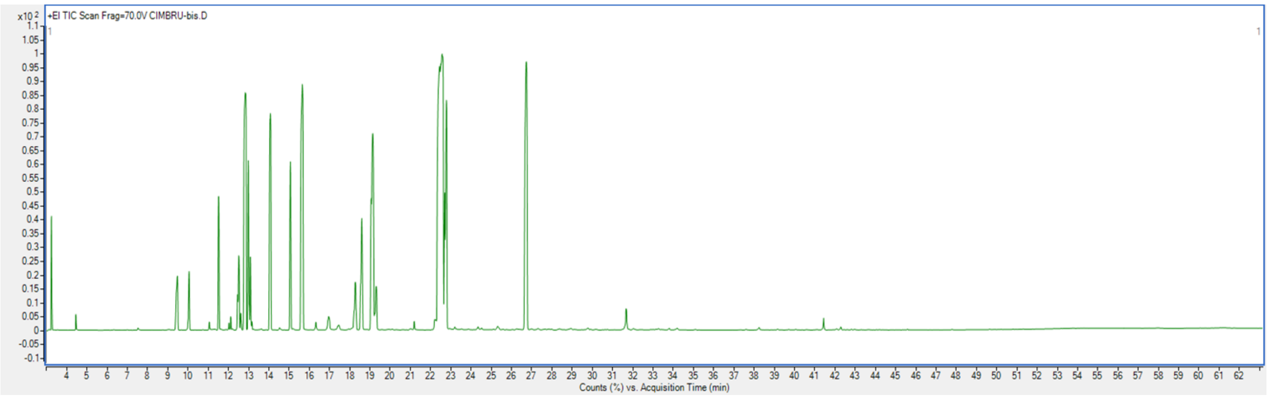

Figure S6: Chromatographic profile of the thyme EO.

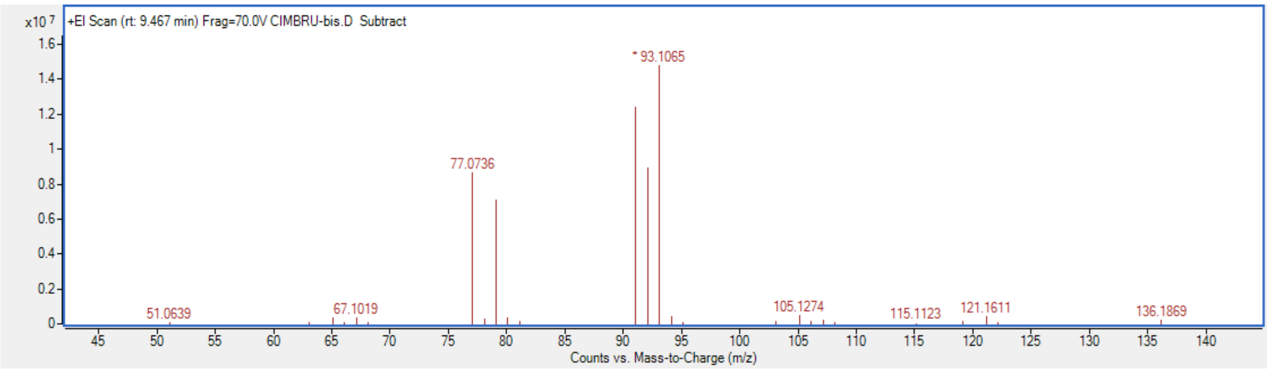

Figure S7.1: Mass spectrum for α-pinene.

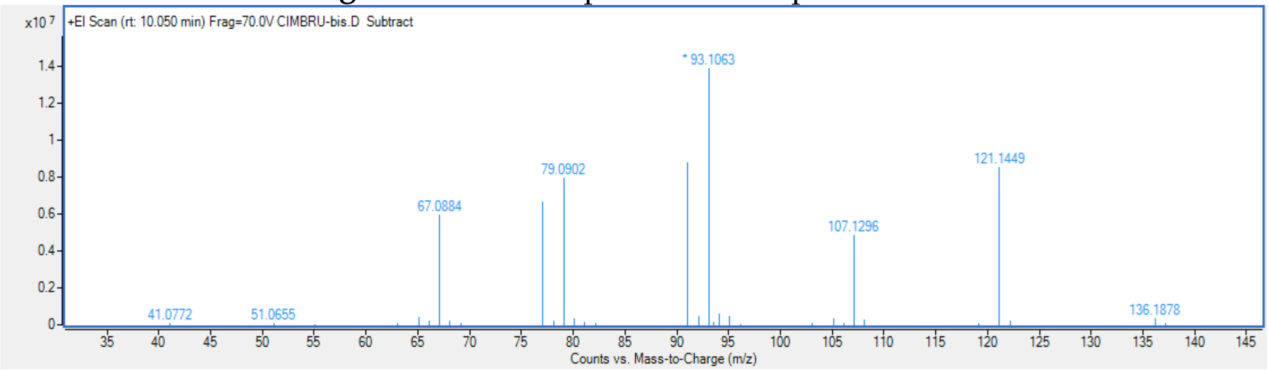

Figure S7.2: Mass spectrum for camphene.

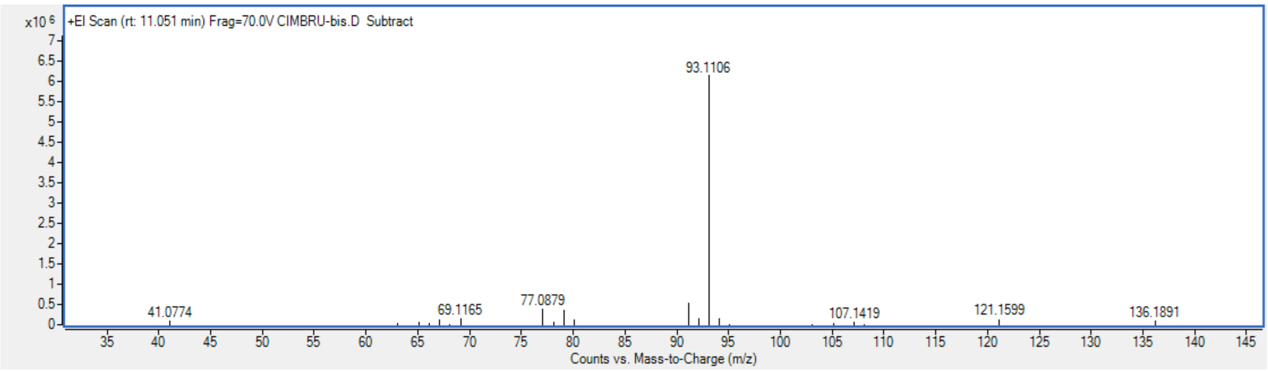

Figure S7.3: Mass spectrum for sabinene.

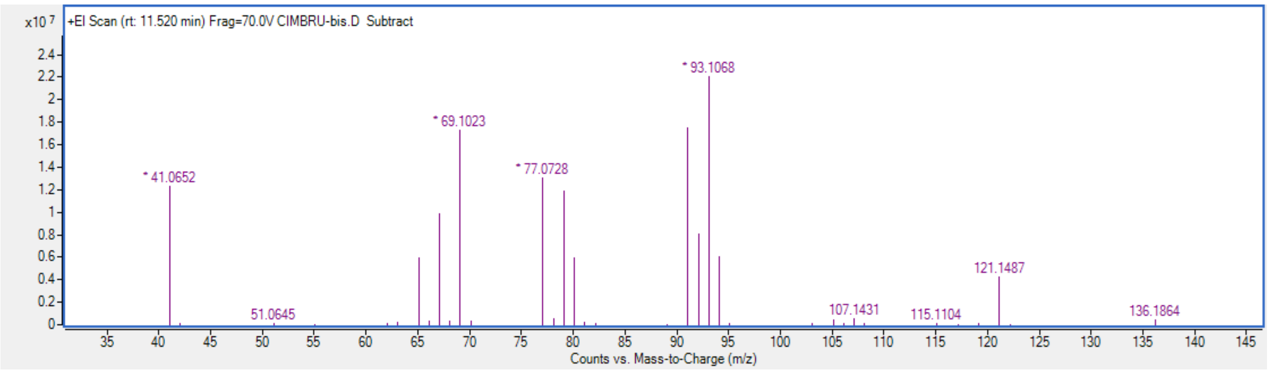

Figure S7.4: Mass spectrum for β-pinene.

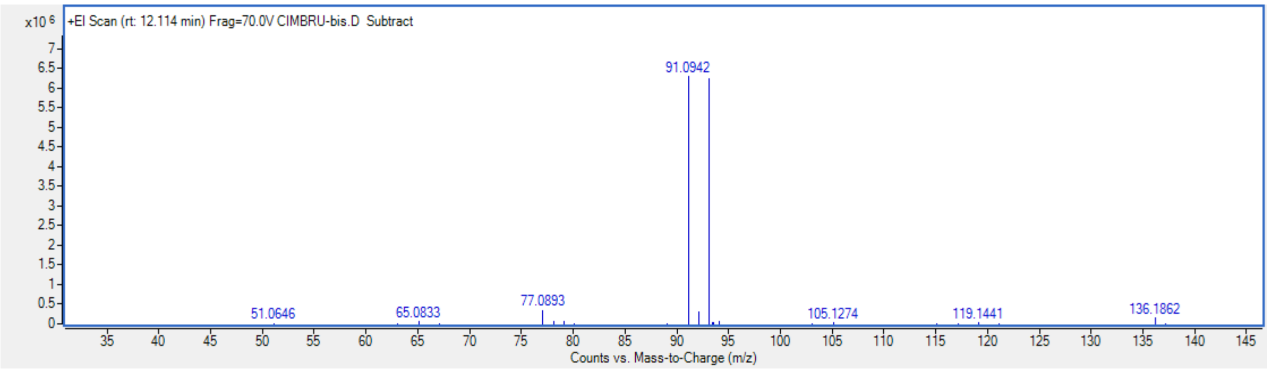

Figure S7.5: Mass spectrum for α-phellandrene.

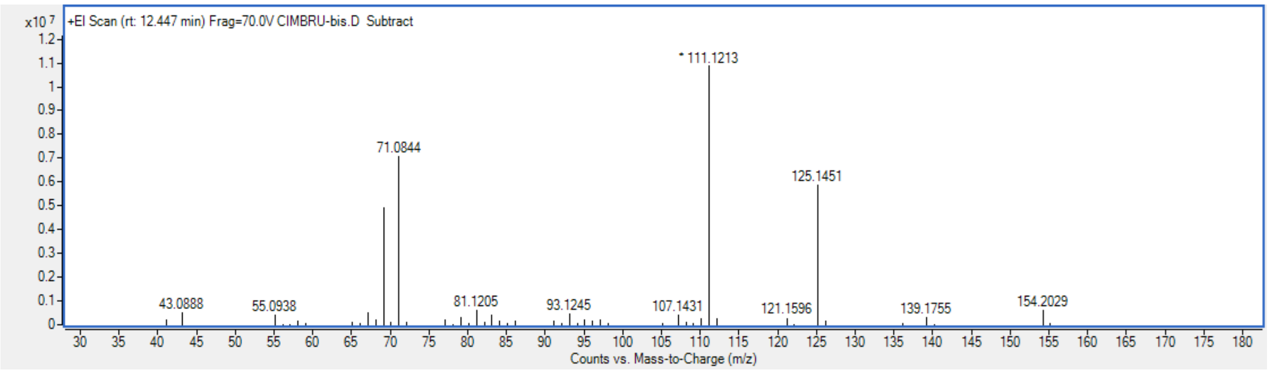

Figure S7.6: Mass spectrum for 1,4-cineol.

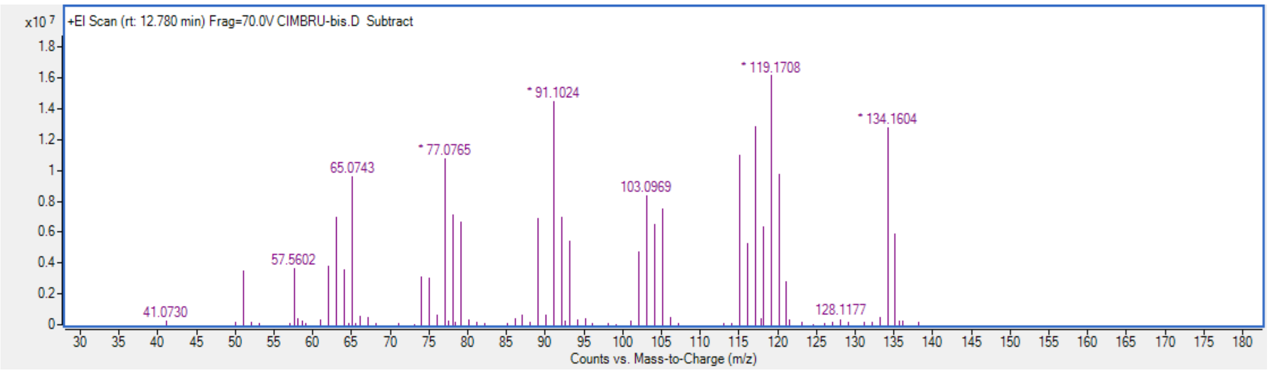

Figure S7.7: Mass spectrum for p-mentha-1,5,8-triene.

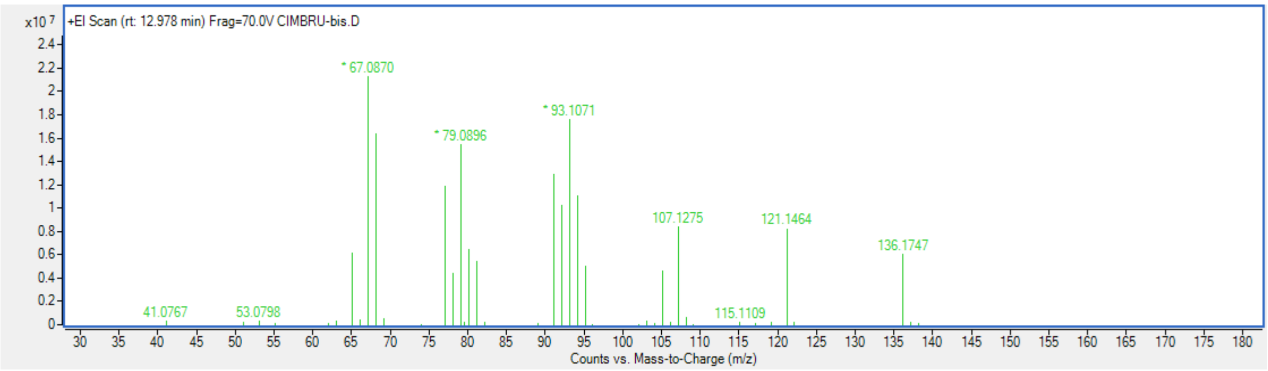

Figure S7.8: Mass spectrum for D-limonene.

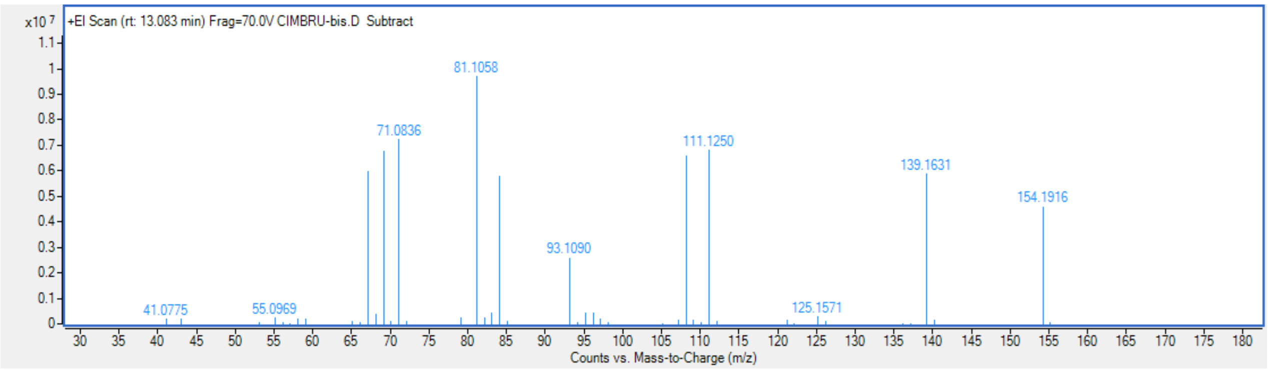

Figure S7.9: Mass spectrum for eucalyptol.

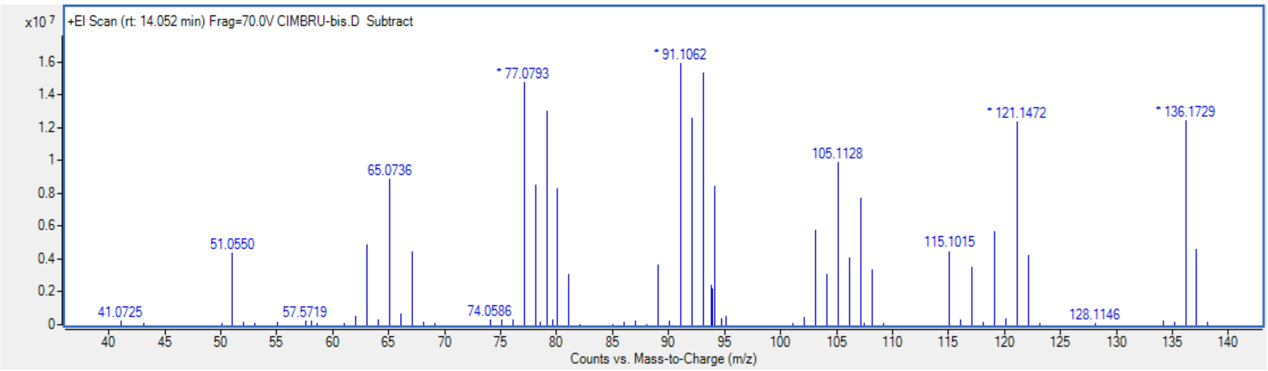

Figure S7.10: Mass spectrum for γ-terpinene.

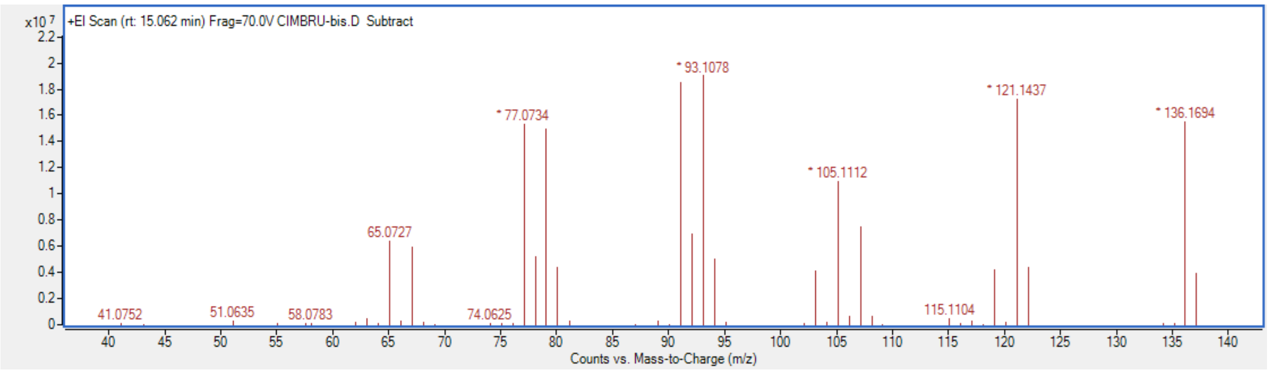

Figure S7.11: Mass spectrum for terpinolene.

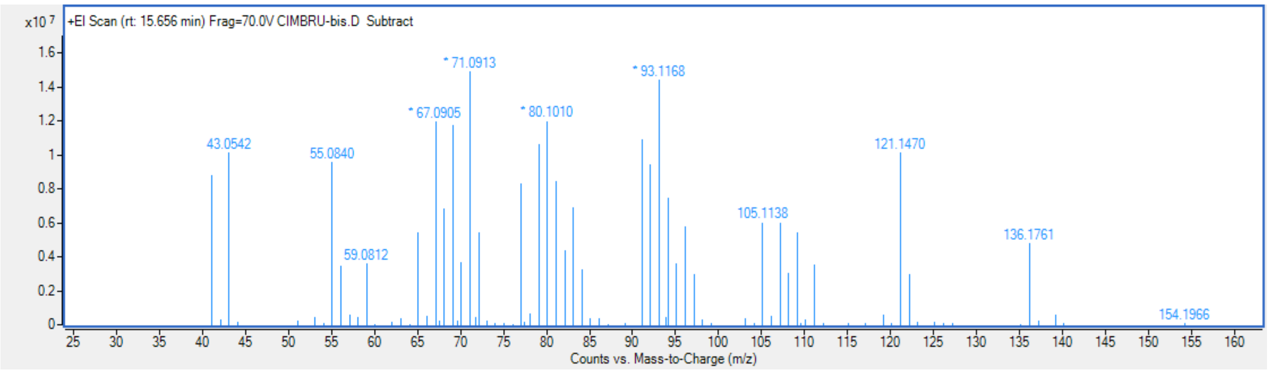

Figure S7.12: Mass spectrum for linalool.

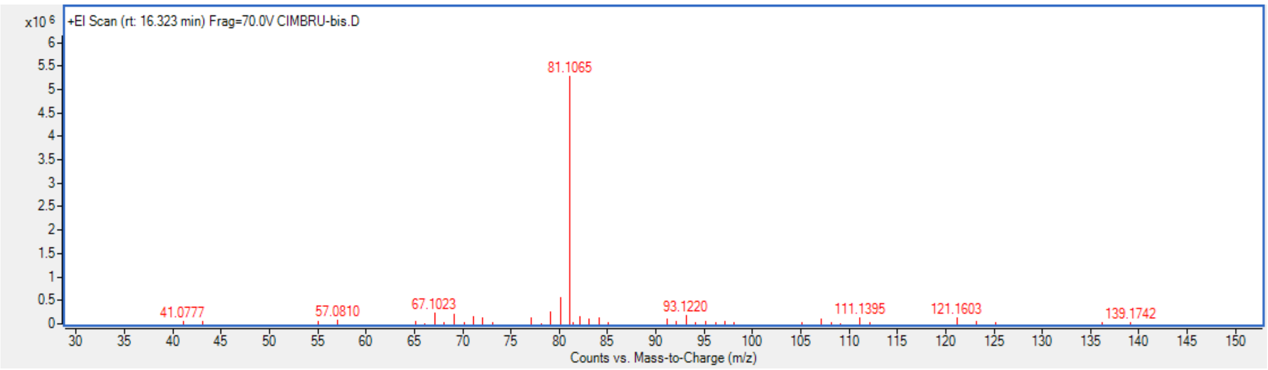

Figure S7.13: Mass spectrum for fenchol.

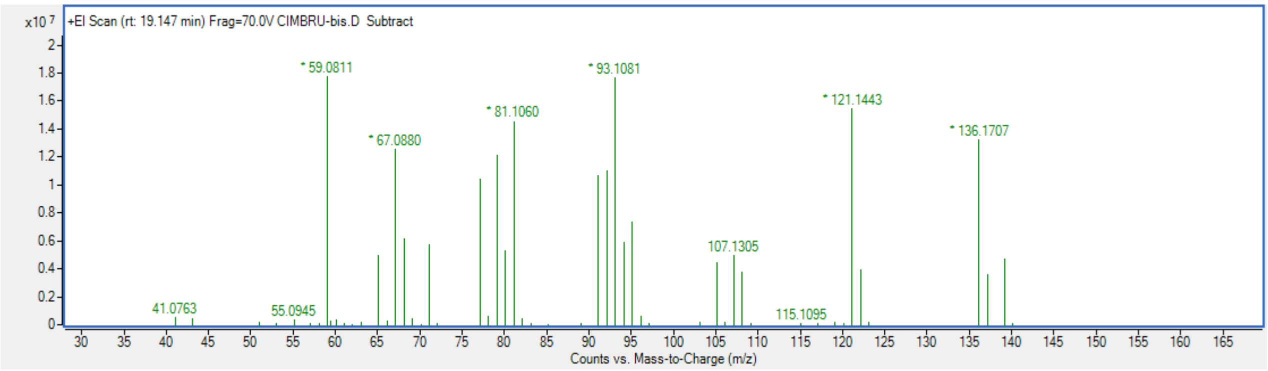

Figure S7.14: Mass spectrum for 1-terpineol.

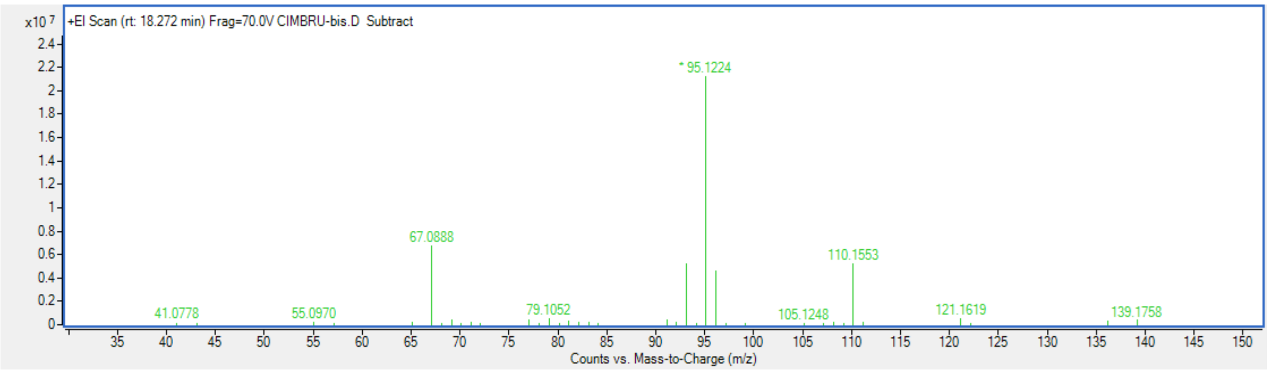

Figure S7.15: Mass spectrum for endo-borneol.

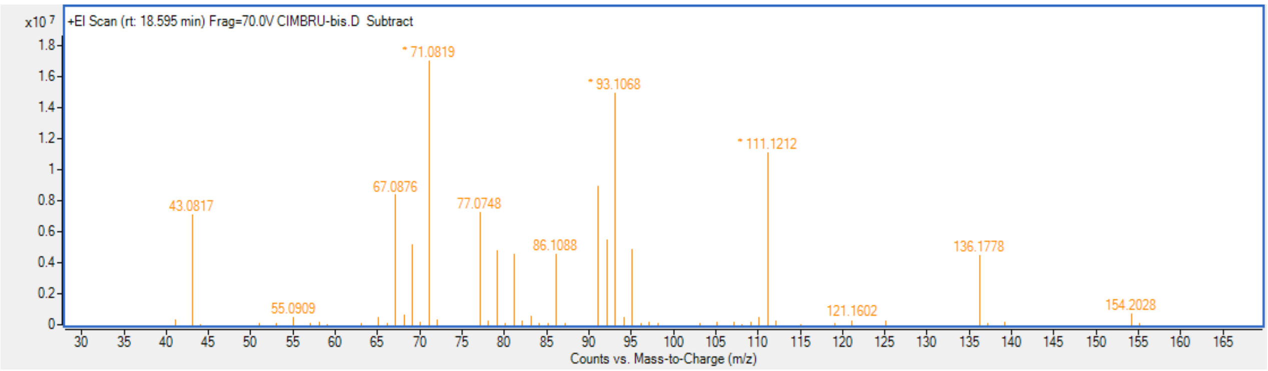

Figure S7.16: Mass spectrum for 4-terpineol.

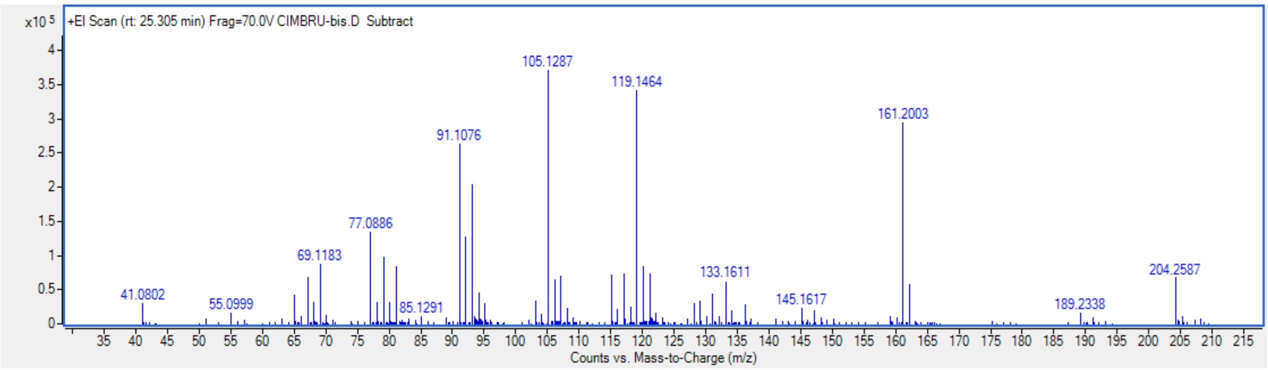

Figure S7.17: Mass spectrum for α-copaene.

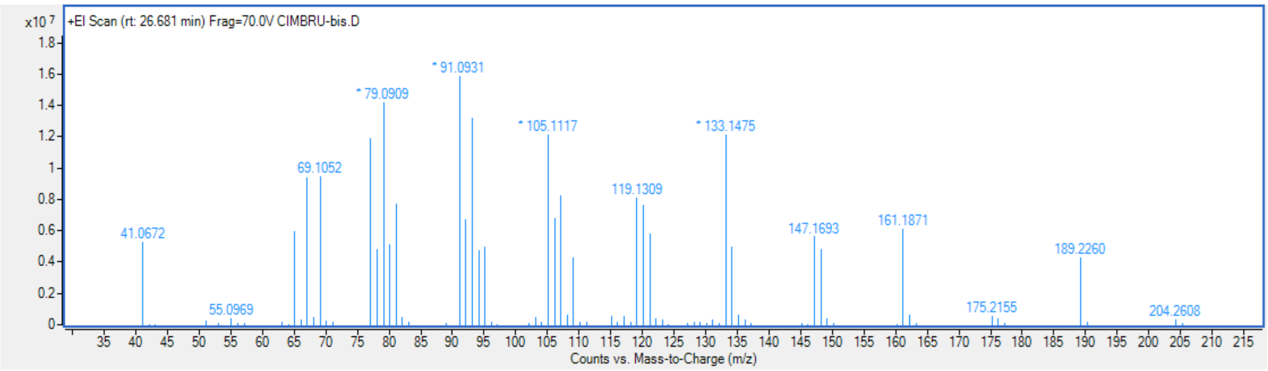

Figure S7.18: Mass spectrum for caryophyllene.

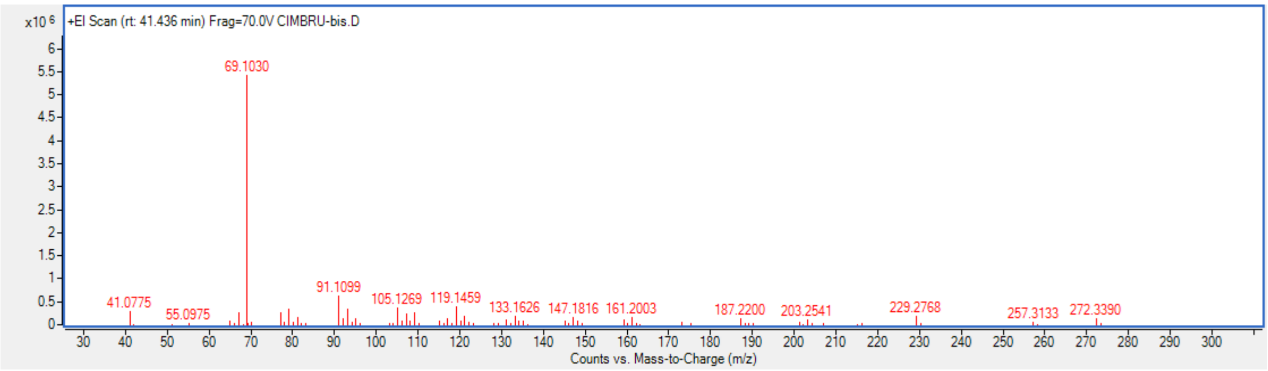

Figure S7.19: Mass spectrum for m-camphorene.

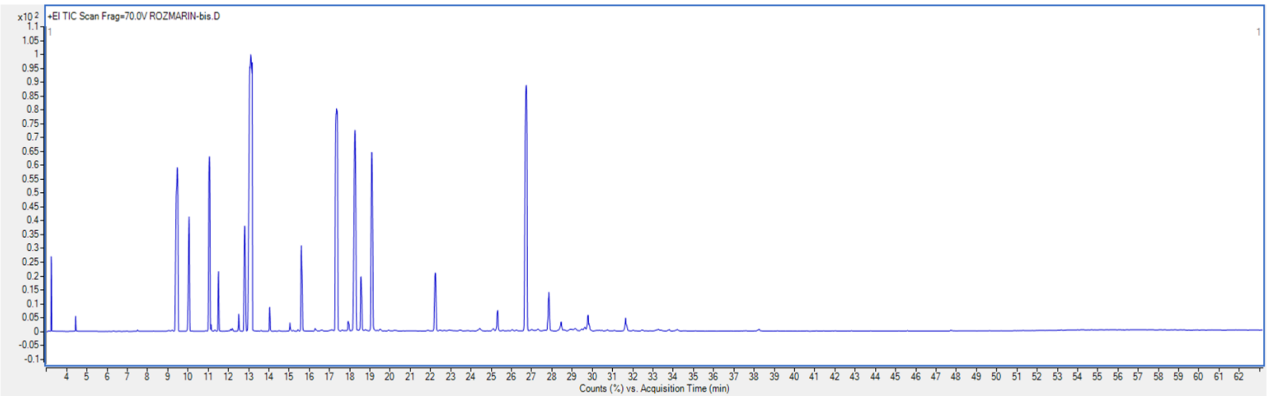

Figure S8: Chromatographic profile of the rosemary EO.

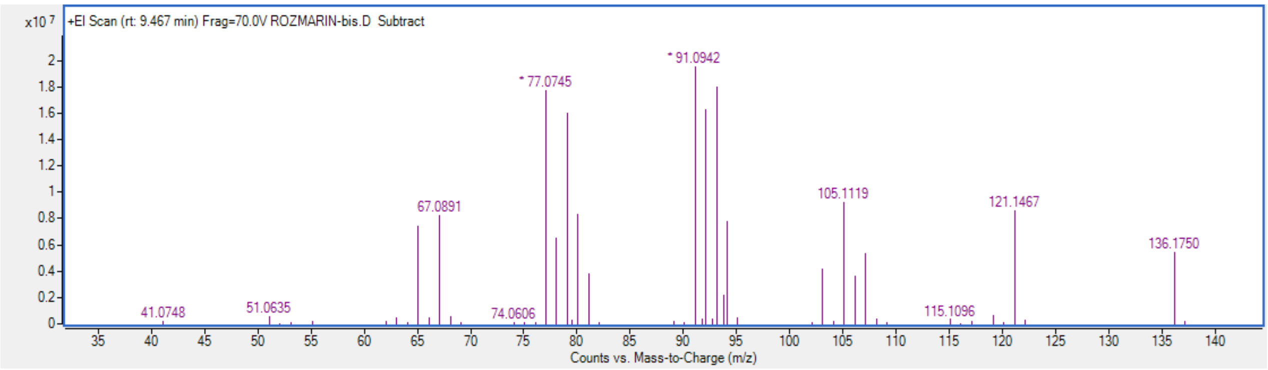

Figure S9.1: Mass spectrum for 3-carene.

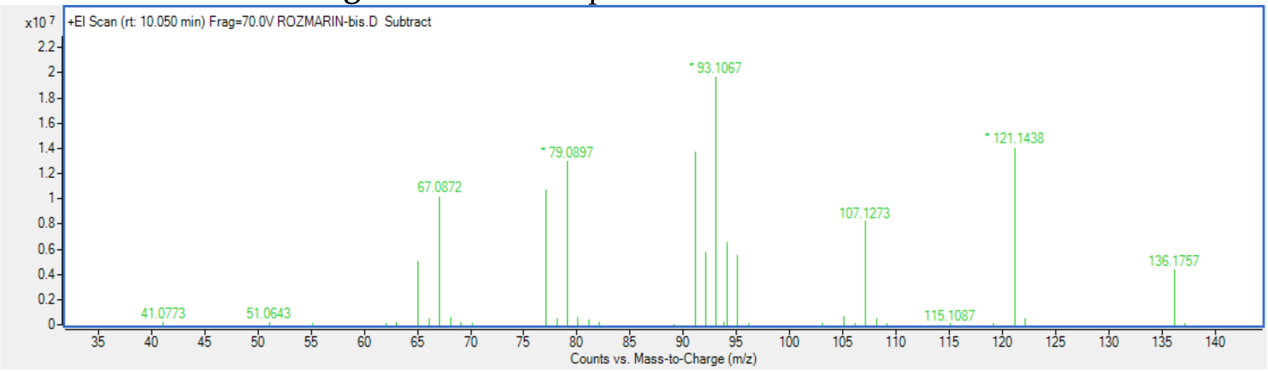

Figure S9.2: Mass spectrum for camphene.

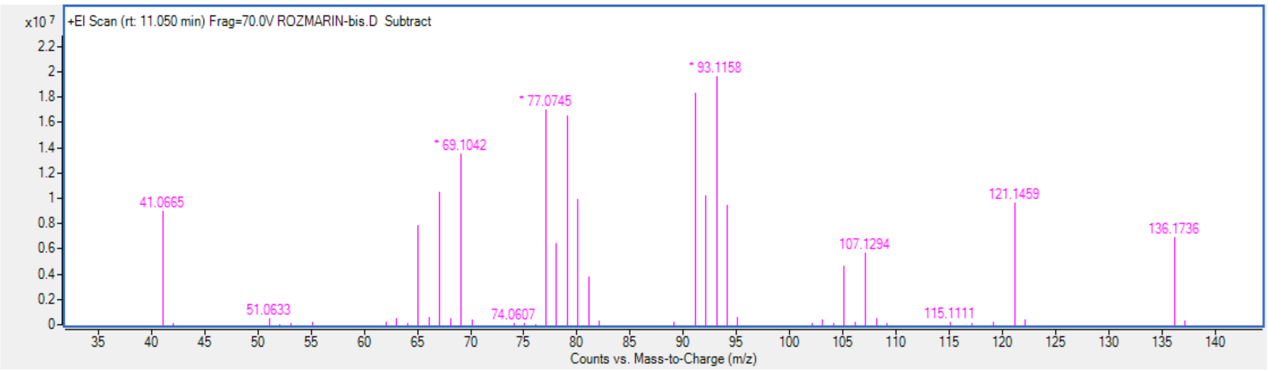

Figure S9.3: Mass spectrum for fenchene.

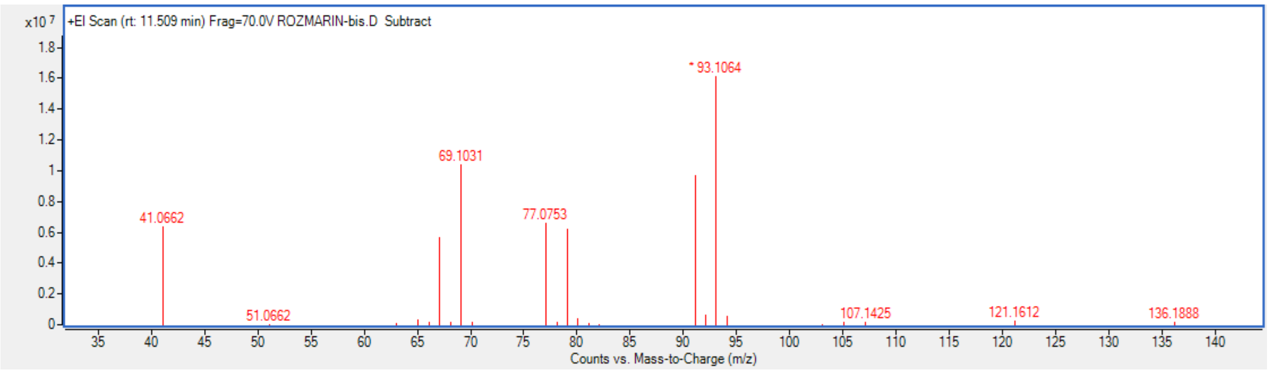

Figure S9.4: Mass spectrum for β-myrcene.

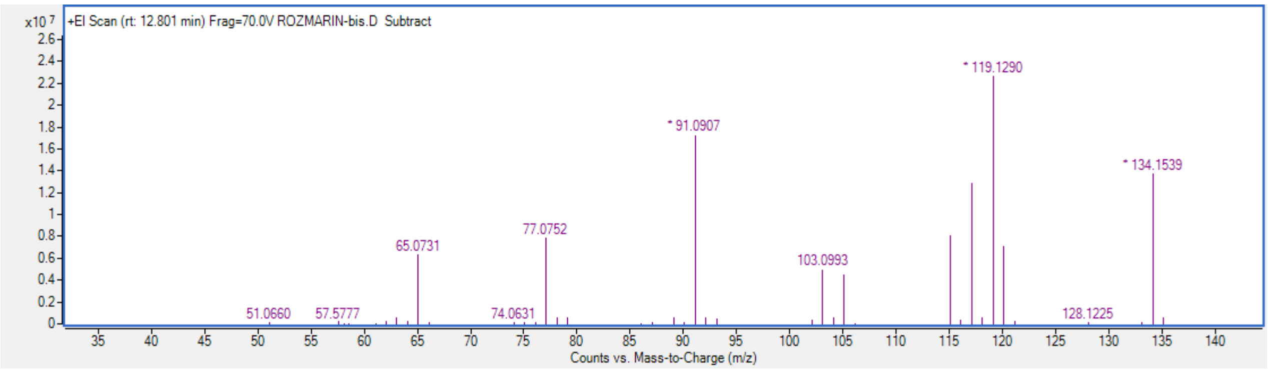

Figure S9.5: Mass spectrum for o-cymene.

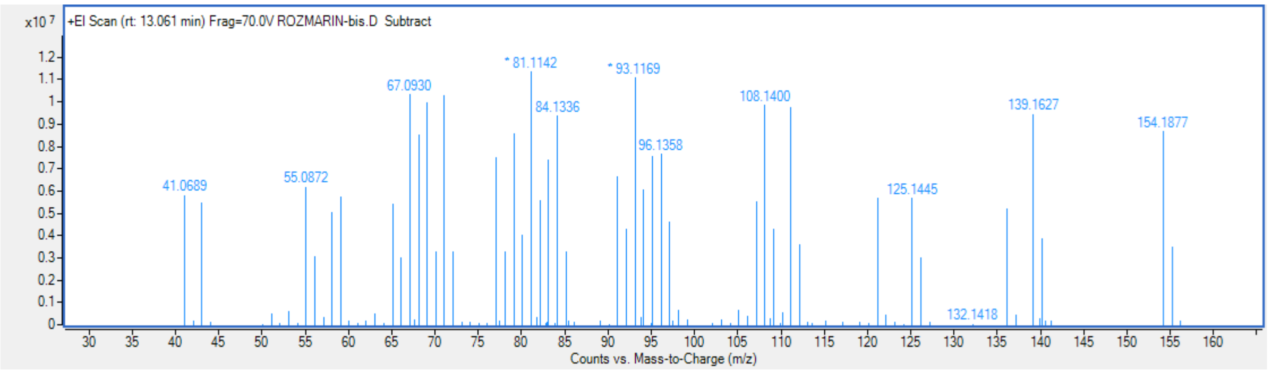

Figure S9.6: Mass spectrum for eucalyptol.

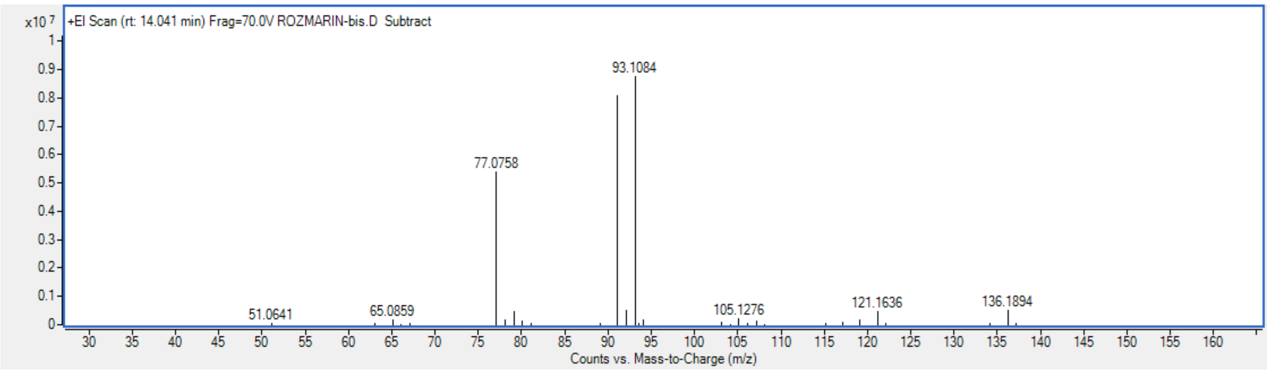

Figure S9.7: Mass spectrum for 3-thujene.

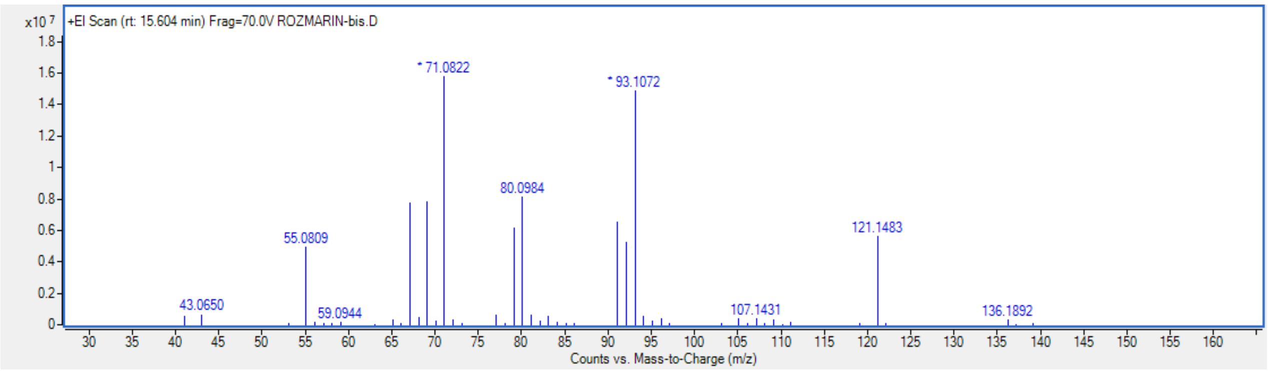

Figure S9.8: Mass spectrum for linalool.

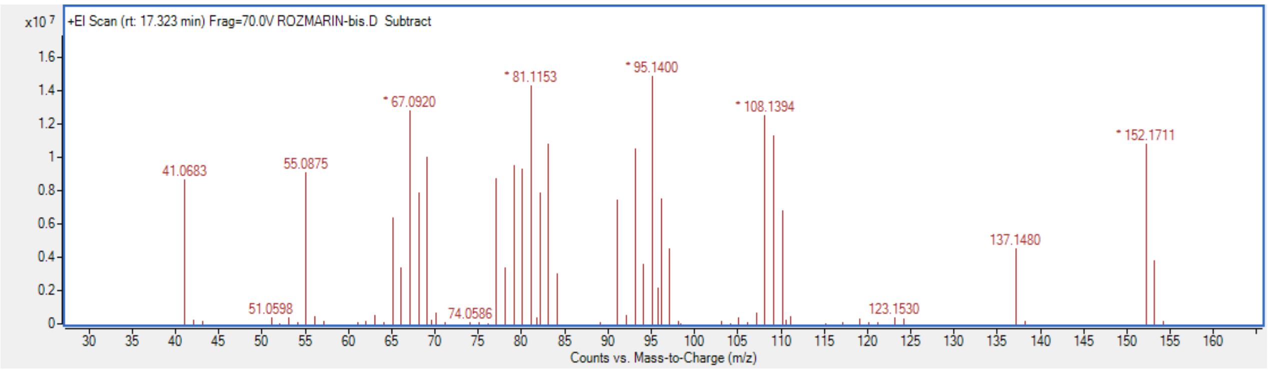

Figure S9.9: Mass spectrum for (+)-2-bornanone.

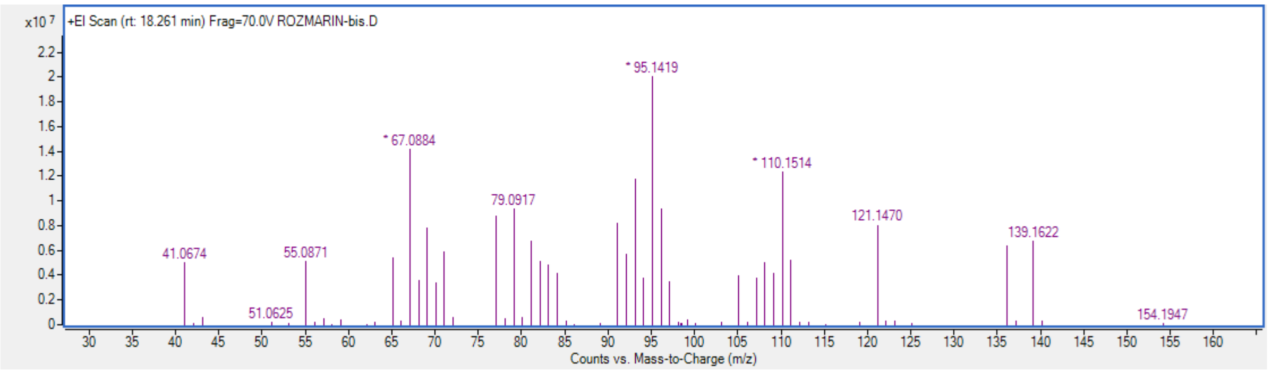

Figure S9.10: Mass spectrum for endo-borneol.

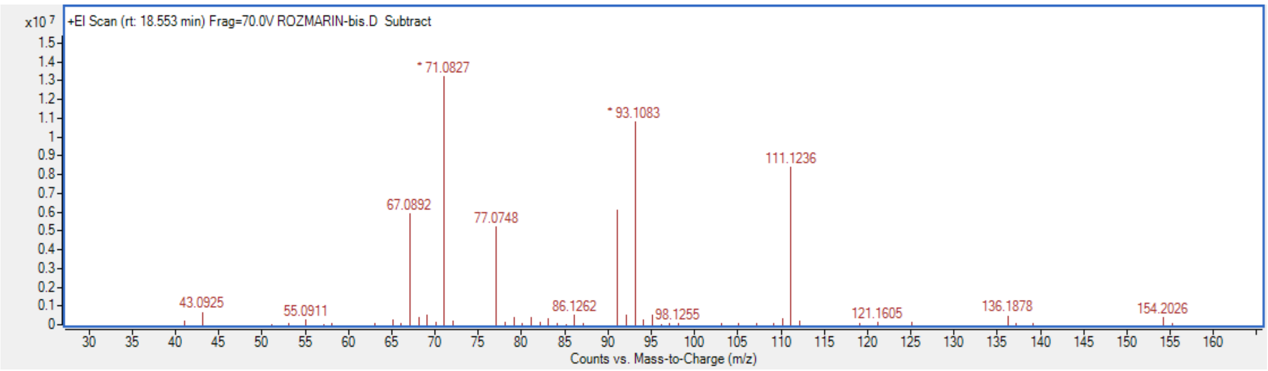

Figure S9.11: Mass spectrum for (-)-4-terpineol.

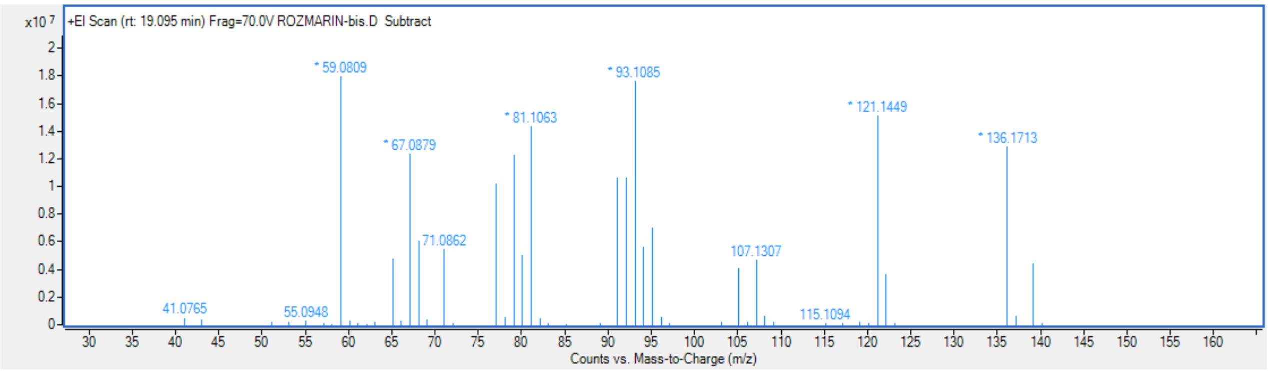

Figure S9.12: Mass spectrum for α-terpineol.

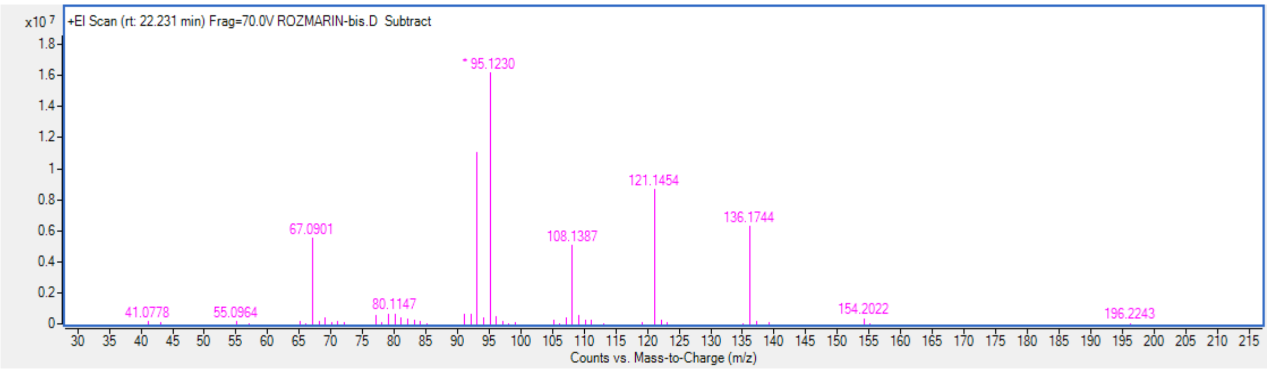

Figure S9.13: Mass spectrum for (-)-bornyl acetate.

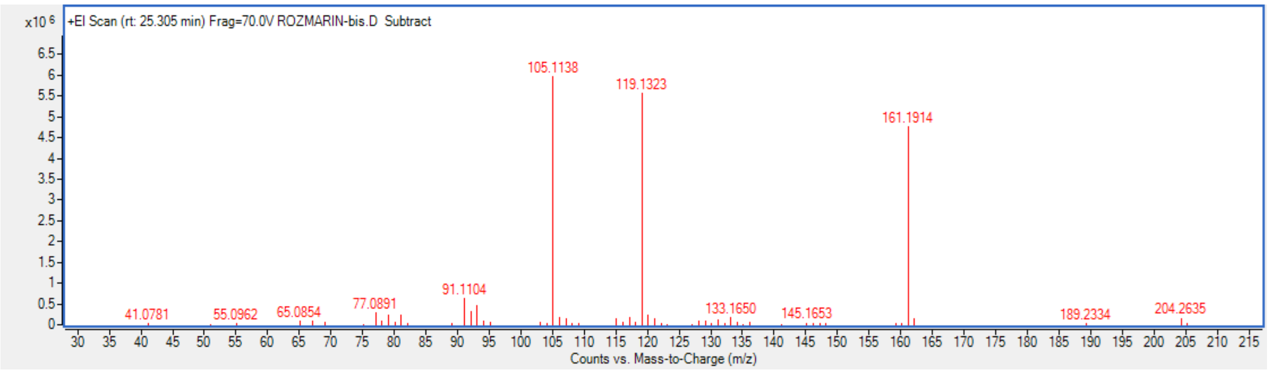

Figure S9.14: Mass spectrum for α-copaene.

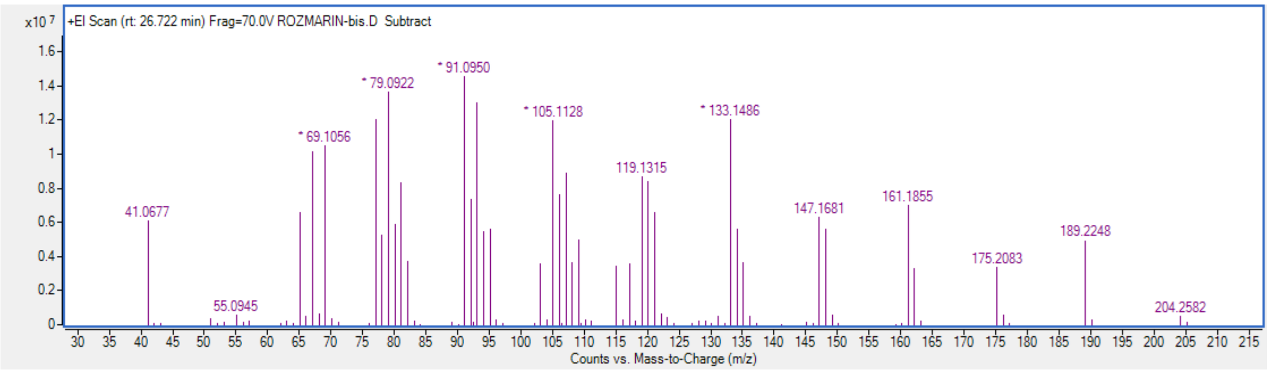

Figure S9.15: Mass spectrum for caryophyllene.

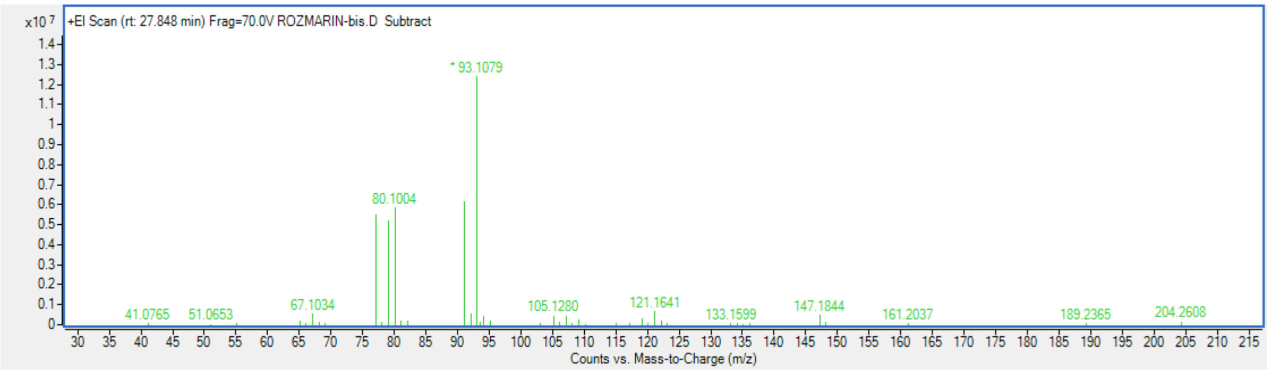

Figure S9.16: Mass spectrum for humulene.

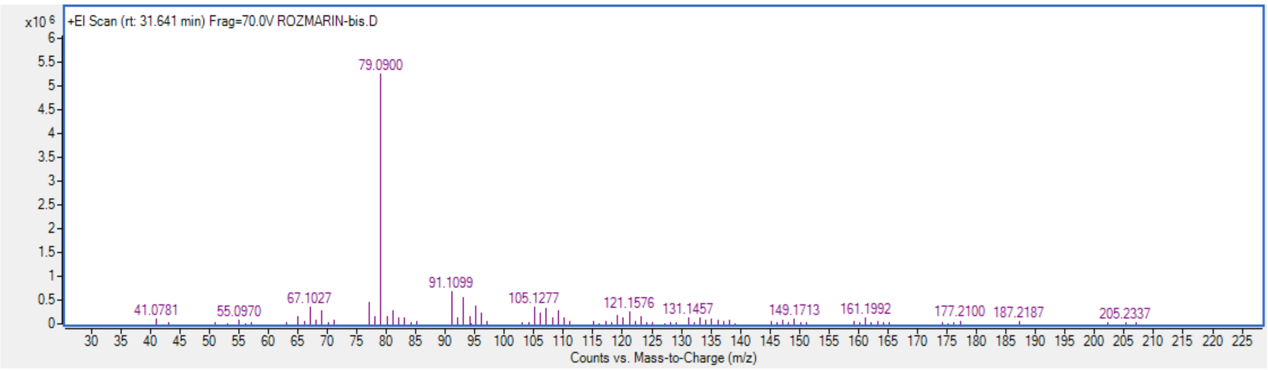

Figure S9.17: Mass spectrum for caryophyllene oxide.

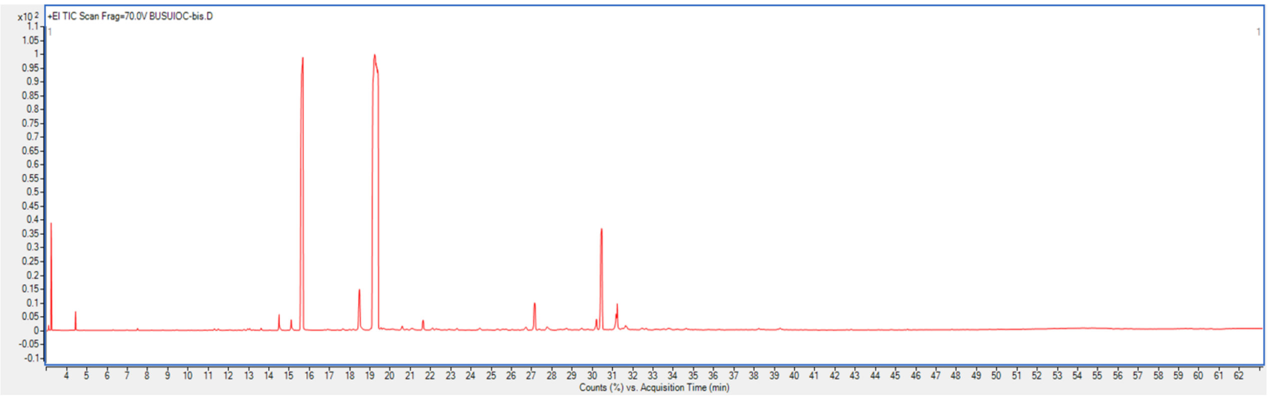

Figure S10: Chromatographic profile of the basil EO.

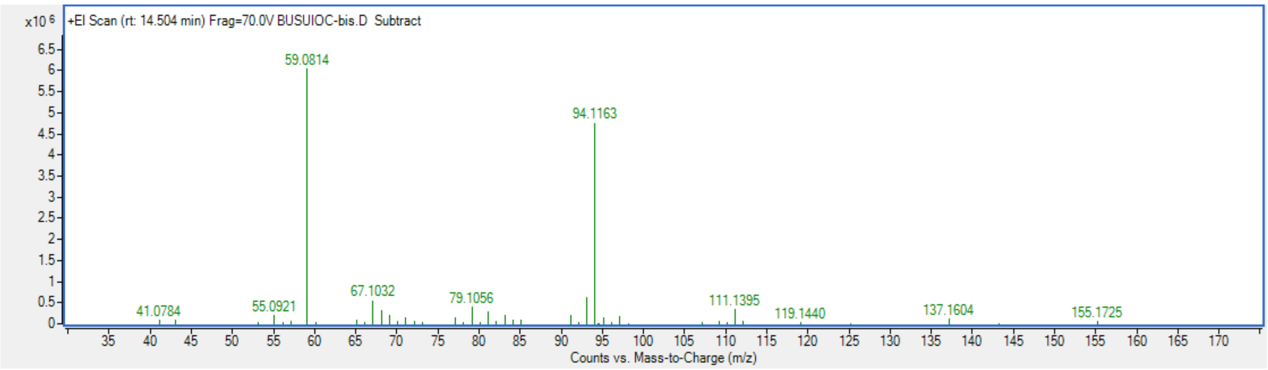

Figure S11.1: Mass spectrum for cis-linalool oxide.

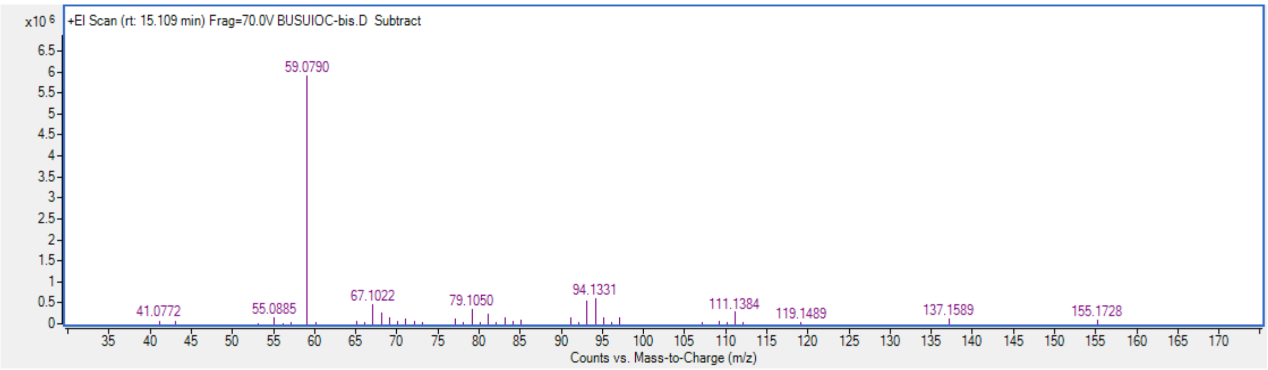

Figure S11.2: Mass spectrum for trans-linalool oxide.

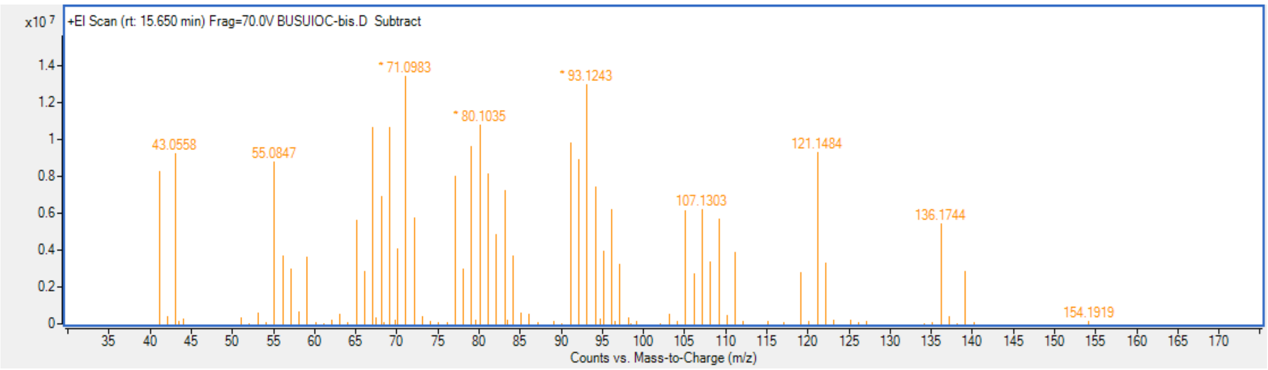

Figure S11.3: Mass spectrum for linalool.

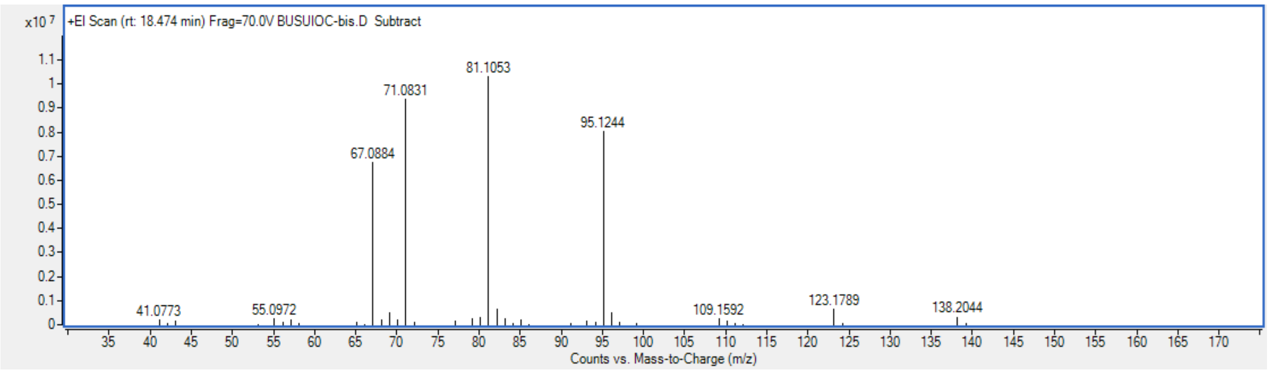

Figure S11.4: Mass spectrum for DL-menthol.

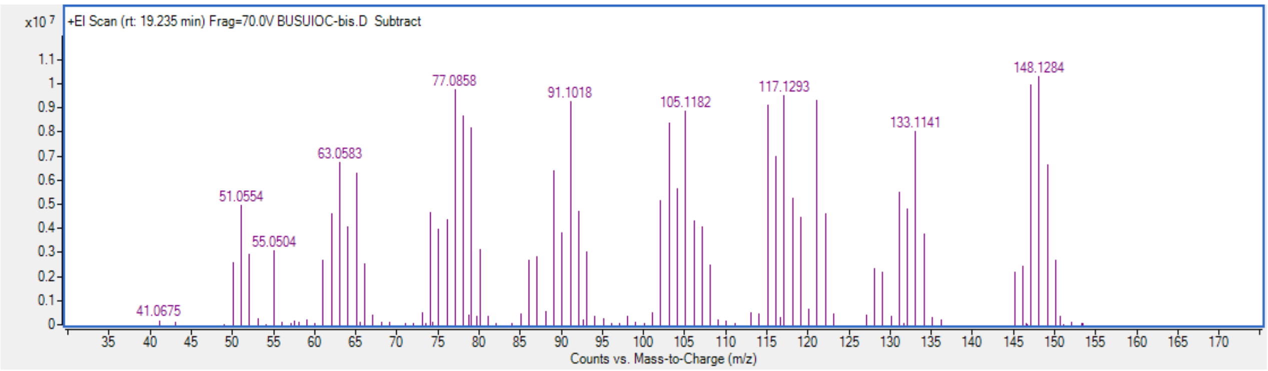

Figure S11.5: Mass spectrum for estragole.

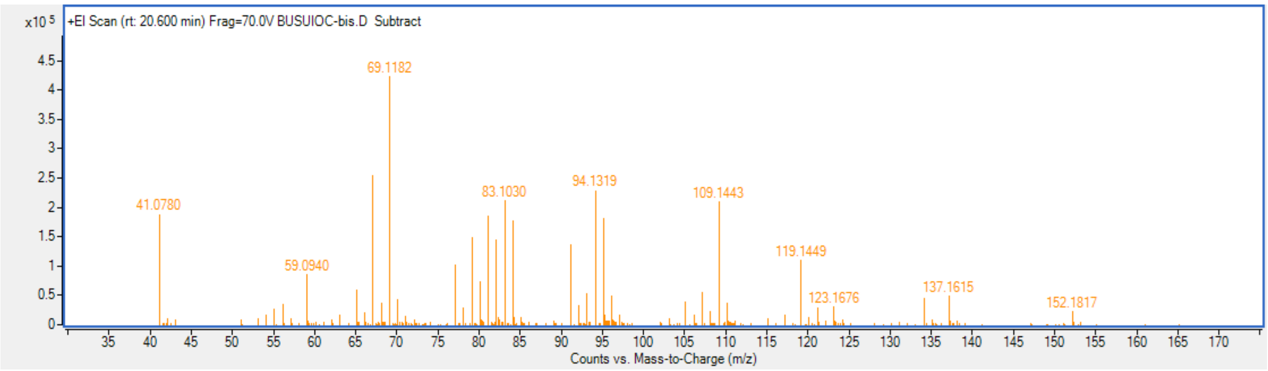

Figure S11.6: Mass spectrum for β-citral.

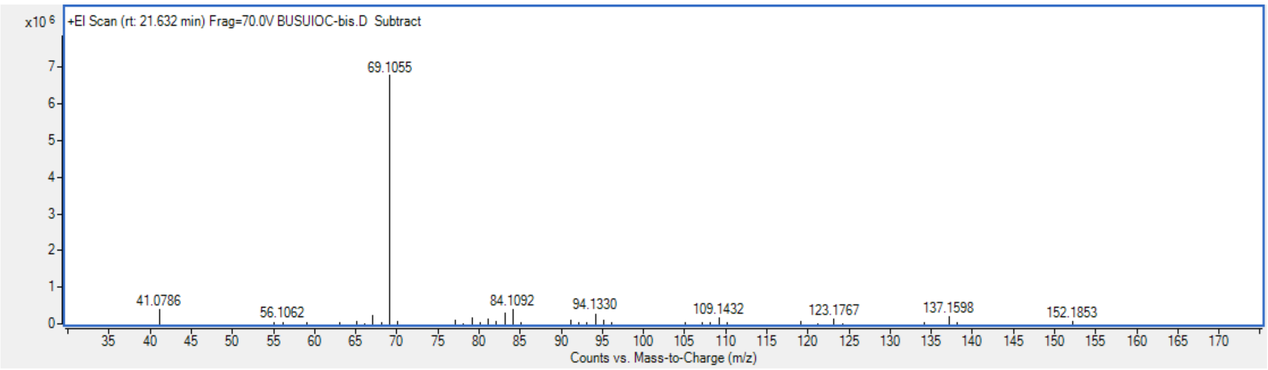

Figure S11.7: Mass spectrum for citral.

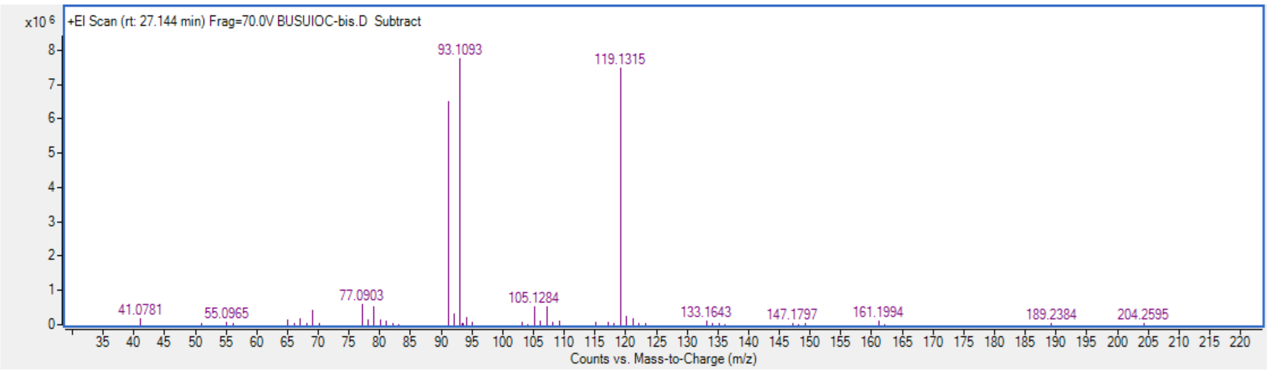

Figure S11.8: Mass spectrum for trans-α-bergamotene.

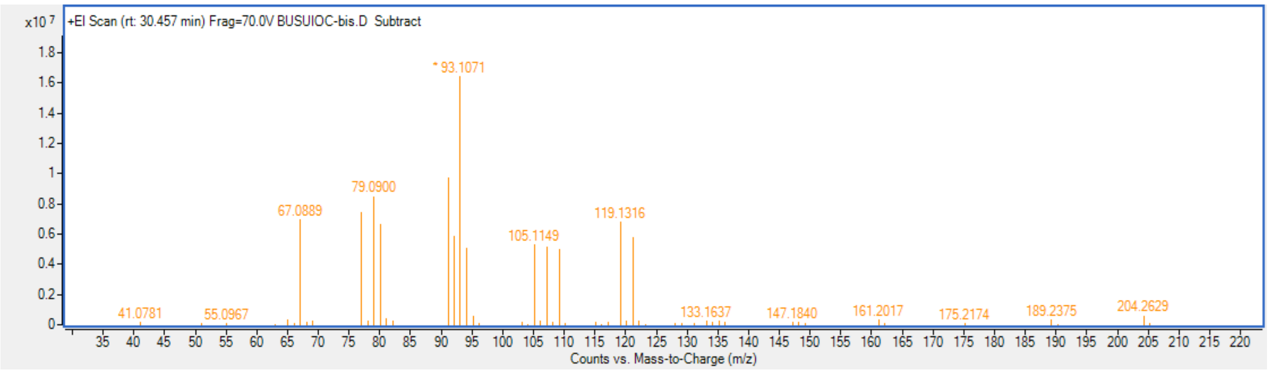

Figure S11.9: Mass spectrum for trans-α-bisabolene.

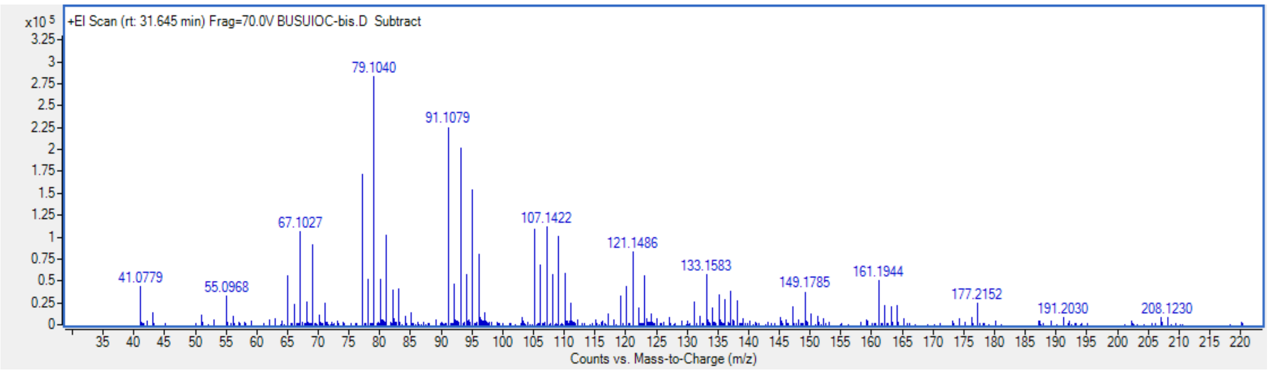

Figure S11.10: Mass spectrum for caryophyllene oxide.

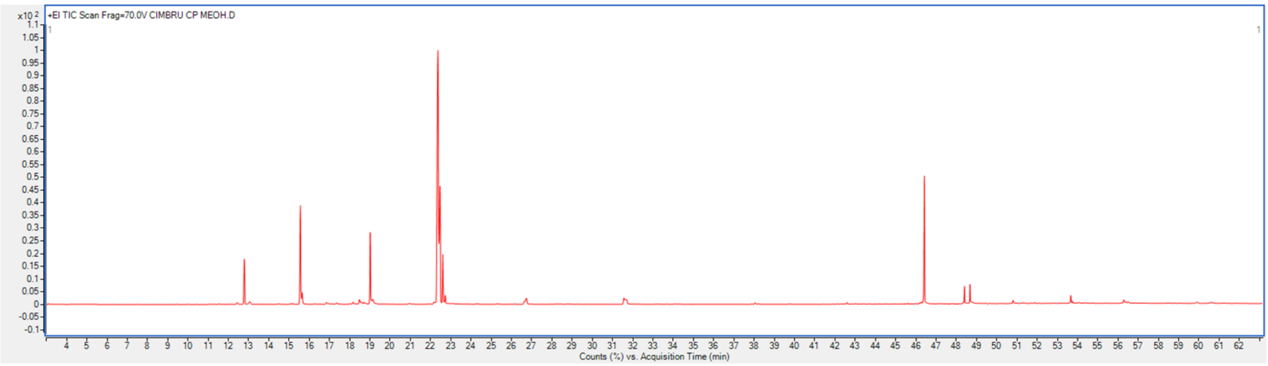

Figure S12: Chromatographic profile for Fe_3O_4@SiO_2@thyme_CP nanosystem (methanol).

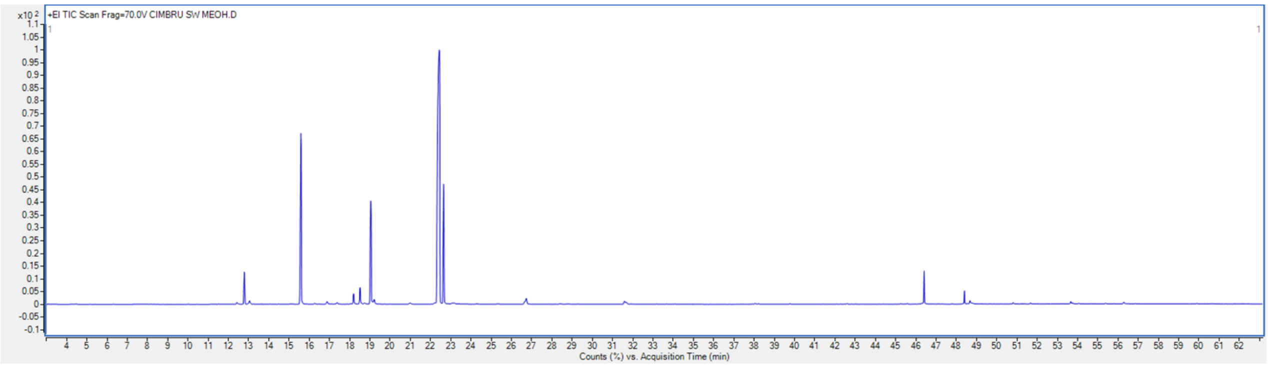

Figure S13: Chromatographic profile for Fe_3O_4@SiO_2@thyme_SW nanosystem (methanol).

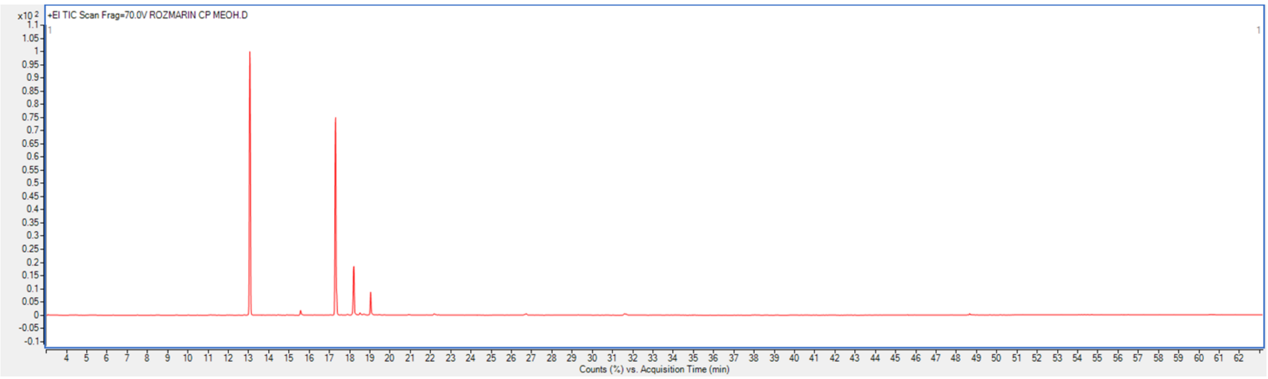

Figure S14: Chromatographic profile for Fe_3O_4@SiO_2@rosemary_CP nanosystem (methanol).

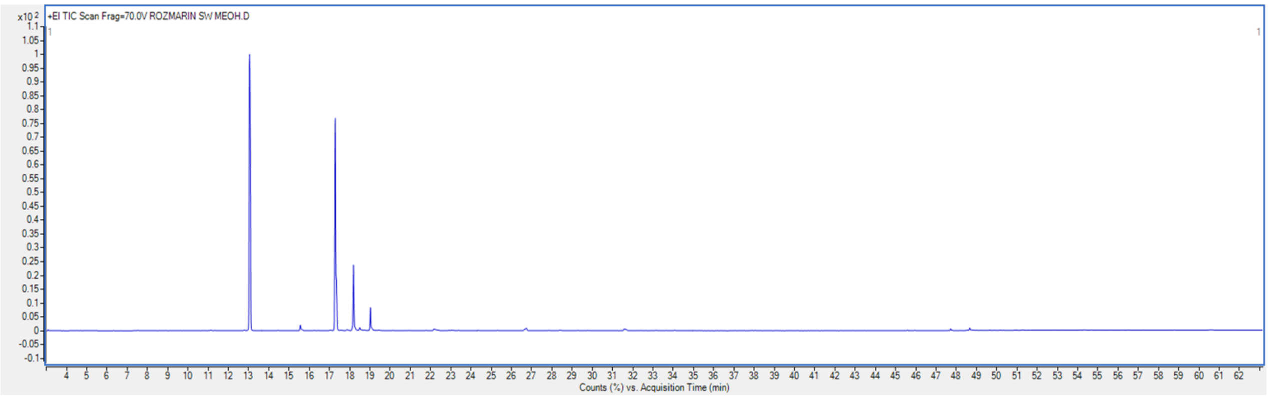

Figure S15: Chromatographic profile for Fe_3O_4@SiO_2@rosemary_SW nanosystem (methanol).

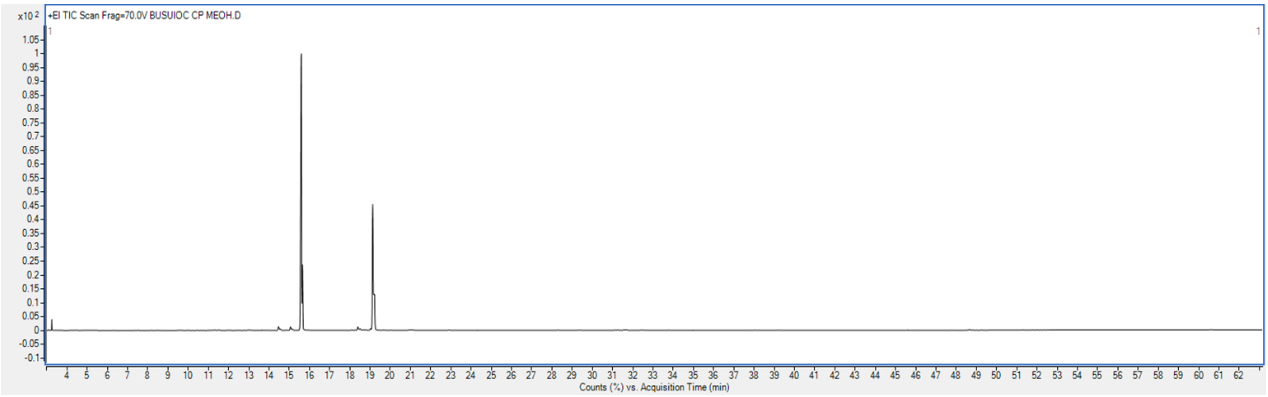

Figure S16: Chromatographic profile for Fe_3O_4@SiO_2@basil_CP nanosystem (methanol).

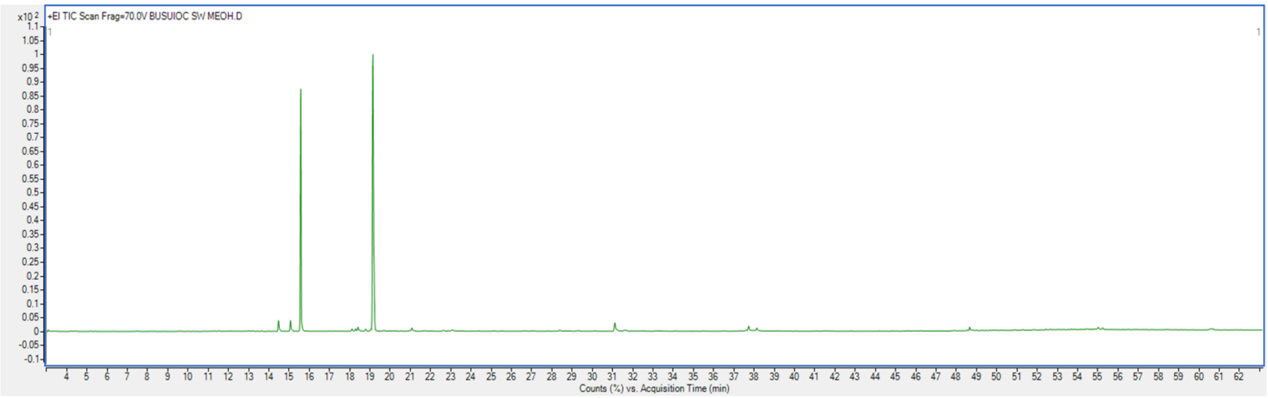

Figure S17: Chromatographic profile for Fe_3O_4@SiO_2@basil_SW nanosystem (methanol).
